# A Rare Complication of Extracorporeal Shock Wave Lithotripsy: Intrarenal Hematoma Mimicking Pelvis Renalis Tumor

**DOI:** 10.1155/2015/719618

**Published:** 2015-05-12

**Authors:** Fatih Akbulut, Onur Kucuktopcu, Burak Ucpinar, Metin Savun, Faruk Ozgor, Erkan Sonmezay, Abdulmuttalip Simsek, Gokhan Gurbuz

**Affiliations:** Department of Urology, Haseki Training and Research Hospital, 34096 Istanbul, Turkey

## Abstract

Extracorporeal shock wave lithotripsy (SWL) is a very commonly used treatment modality for appropriate sized stones. Even though it is a noninvasive treatment technique, major complications may occur following SWL sessions. Herein, we report a 17-year-old male patient, who received 2 sessions of SWL treatment for his left kidney stone, 4 months before his admission. Imaging methods showed an enhanced left renal pelvis mass with contrast-enhanced computerized tomography (CT) and this finding raised a suspicion of pelvis renalis tumor. Diagnostic ureterorenoscopy was planned for the patient and operation revealed a left intrarenal hematoma, which was drained percutaneously during the same operation. Careful history should be taken from patients with renal pelvis masses and intrarenal hematoma formation should be kept in mind, especially if the patient has a previous SWL history.

## 1. Introduction

Extracorporeal shock wave lithotripsy (SWL) is a very important treatment modality for kidney and ureteral stones, since it was first described by Chaussy in Germany, in the 1980s [1]. Because of its low complication rates and high efficacy in stone clearance, it has been the first choice of treatment for kidney stones smaller than 2 cm and ureteral stones smaller than 1 cm [2]. Despite its low complication rates, serious complications can occur after SWL treatment. Symptomatic intrarenal, subcapsular, or perirenal hematomas can be seen in less than 1% of patients. However, if every patient is assessed with imaging methods after SWL treatment, this ratio increases up to 20–25% [3]. In this case report, we aim to present a patient who received 2 sessions of SWL treatment in a different clinic and who was admitted to our clinic with left flank pain 4 months after the SWL sessions and imaging modalities raised a suspicion of renal pelvis tumor.

## 2. Case Report

**Figure 1:**
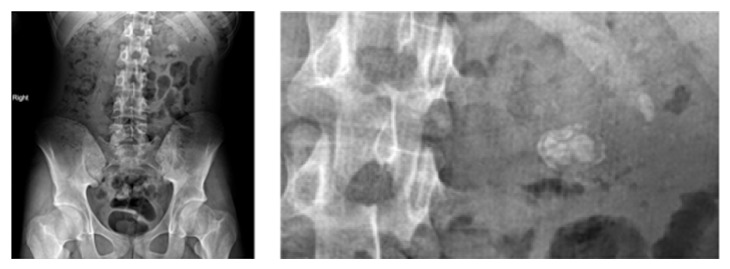
Preoperative KUB radiography of patient.

A 17-year-old male patient was admitted to our outpatient clinic with left flank pain. Four months ago, he received 2 sessions of SWL treatment within 2 weeks, for his left kidney stone. Physical examination was unremarkable. The intravenous pyelography (IVP), which was taken before the SWL sessions, shows 22∗14 mm opacity in left renal pelvis and grade 3 hydronephrosis. Dimercaptosuccinic acid (DMSA) scan showed a relative function of 19% in the left kidney, before the SWL treatment. Kidney, ureter, and bladder X-ray (KUB radiography) on admission showed 2 cm of disorganized opacity around lumbar 1-2 vertebral levels (Figure 1).

**Figure 2:**
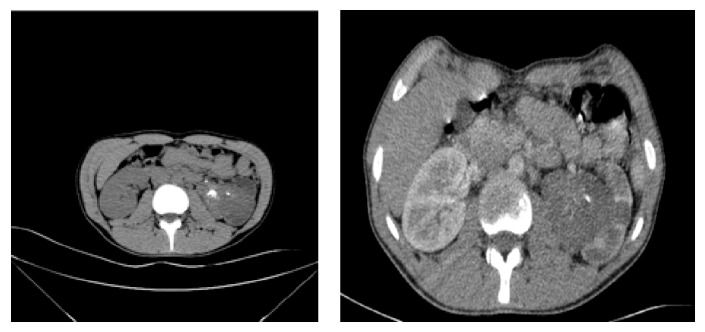
Preoperative CT with and without contrast.

Noncontrast and contrast-enhanced computerized tomography (CT) revealed 6∗5 cm of solid mass with calcifications within the left renal pelvis and lymph nodes up to 2 cm in diameter, in left para-aortic area. These findings raised a suspicion of renal pelvis tumor (Figure 2). Laboratory tests results were Hb: 13 g/dL; WBC: 6900/uL; PLT: 195000/uL; Cr: 0.6 mg/dL; Ca: 10.1 mg/dL; PT: 1.25 sec.; INR: 1.05; APTT: 27,5 sec. Complete urine analyses were within normal limits and urine culture was sterile.

**Figure 3:**
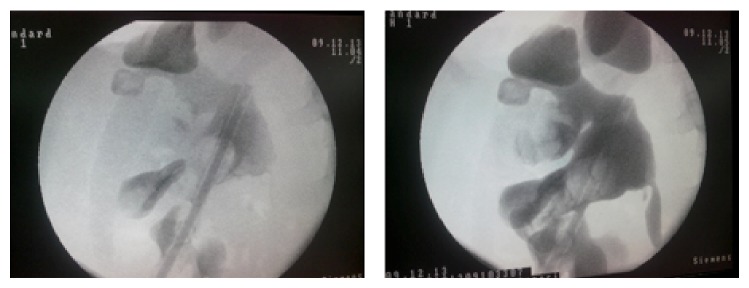
Postoperative second day nephrostography showed passage through ureter and no extravasation.

Left diagnostic ureterorenoscopy (URS) was planned for the patient, due to the suspicion of left renal pelvis tumor. In lithotomy position, 9,5 Fr ureterorenoscope was used for diagnostic URS. Organized hematoma filling the renal pelvis was identified. 4 Fr ureteral catheter was inserted and patient was positioned to prone position for percutaneous drainage of the hematoma. Lower calyceal system was dilated by 30 Fr balloon dilatators and hematoma and stone fragments were cleared by using ultrasonic lithotripter (EMS Swiss LithoClast (R) Master) and basket catheter (NCircle Tipless Stone Extractor Cook Medical). 14 Fr nephrostomy tube was inserted at the end of the operation. Operation time was 140 minutes and no bleeding occurred during the operation. On first postoperative day, Foley and ureteral catheter were removed and on second postoperative day nephrostomy tube was removed after confirming that complete stone clearance was achieved and there was no extravasation in antegrade nephrostography (Figure 3).

## 3. Discussion

The idea of using focused shock waves arose in the early 1970s and trials were made by in vitro methods and animal trials. First human trial of SWL was performed in February 1980 by Chaussy using Dornier HM 1 [4]. In 1983 Dornier HM 3 was introduced. Although SWL is an effective and less invasive method, when compared to surgery, in treating kidney and ureteral stones, SWL related complications can not be ignored and should be kept in mind. Presence of pregnancy, uncontrolled urinary tract infection, bleeding tendency, uncontrolled hypertension, aortic and renal artery aneurysms, serious skeletal malformations, and morbid obesity are contraindications for SWL treatment [5].

Complications of SWL can be classified as early and late complications. Early complications can be subdivided as complications related to stone fragments, infections, and tissue damage. Tissue damage can result in hemorrhage and hematoma formation in kidney and can cause trauma to cardiovascular, gastrointestinal, and genital systems and fetus. Late complications are decreased renal function, hypertension, and complications about fertility [6]. In our case we determined an early, undiagnosed, rarely seen, and intrarenal hematoma formation.

The type of lithotripter can affect the hematoma formation. More energy is delivered to the kidney in electrohydraulic shock waves and this feature of electrohydrolic shock waves can cause more trauma than electromagnetic energy [7]. In a study consisting of 570 patients who have received SWL treatment by using electromagnetic energy source, age was the only statistically significant variable on hematoma formation [4].

Symptomatic fluid collections and perirenal, subcapsular, or intrarenal hematoma formations after SWL treatment are rare complications but if asymptomatic patients are evaluated by CT or magnetic resonance imaging (MRI) this ratio increases up to 25% [3]. In a study by Navarro et al., between 1992 and 2007, among the 4815 patients who have received SWL treatment, serious subcapsular and perirenal hematoma formation was identified only in 7 patients (1%) [8]. In a radiological evaluation study, Rubin et al. evaluated 50 patients with CT, before and after SWL treatment. Subcapsular hematoma was identified in 8 patients (15%) and intrarenal hematoma was identified in 2 (4%) patients [9]. Since Engel and Page described hypertension due to compression of kidney by perirenal hematoma, this condition is called “Page kidney.” Treatment of page kidneys consists of decreasing compression around the kidney by percutaneous, open, or laparoscopic techniques [10]. In a case report, Tuteja et al. presented ischemic anuria related to bilateral subcapsular hematoma formation after bilateral SWL treatment. Patient was evaluated by cystoscopy and pyelography. Anuria was related to compression ischemia described by Page [11] but not related to obstructive stone or clot fragments [12]. In a case report by Inoue et al., in 2010, 76-year-old male patient could not survive due to the massive retroperitoneal hemorrhage after SWL treatment, despite emergent nephrectomy [13].

To our knowledge, there is no case report reporting such an intrarenal hematoma formation after SWL treatment. Hemorrhage and obstructive stone fragments after SWL sessions may have caused ineffective drainage and caused intrarenal hematoma formation. CT evaluation with and without contrast explained the opacity of stone fragments as calcifications and contrast involvement of renal pelvis tumor. We performed diagnostic URS due to the SWL history of patient. After identifying the intrarenal hematoma, we performed percutaneous removal of hematoma and stone fragments.

## 4. Conclusion 

Even though SWL is a noninvasive technique, it might occasionally result in serious complications. Careful history should be taken from patients with renal pelvis masses and intrarenal hematoma formation should be kept in mind, especially if the patient has a recent SWL history. Thereby, diagnosis of suspicious masses should be verified by diagnostic URS. Percutaneous hematoma drainage and stone removal are a safe treatment method in patients with organized intrarenal hematoma, related to SWL treatment.
